# PD-1/PD-L1 antibodies efficacy and safety versus docetaxel monotherapy in advanced NSCLC patients after first-line treatment option: systems assessment

**DOI:** 10.18632/oncotarget.19641

**Published:** 2017-07-27

**Authors:** Qiang Su, Zhigang Sun, Chenguang Zhang, Yanli Hou, Bangwei Cao

**Affiliations:** ^1^ Department of Oncology, Beijing Friendship Hospital, Capital Medical University, Beijing, China; ^2^ Department of Thoracic Surgery, Jinan Center Hospital Affiliated to Shandong University, Shandong University, Jinan, China; ^3^ Department of Biochemistry and Molecular Biology, School of Basic Medical Sciences, Capital Medical University, Beijing, China; ^4^ Department of Ophthalmology, Beijing Friendship Hospital, Capital Medical University, Beijing, China

**Keywords:** NSCLC, PD-1 antibody, PD-L1 antibody, docetaxel, meta analysis

## Abstract

Meta-analysis was conducted to systematically assess the effectiveness and safety of programmed cell death protein-1 or ligand-1 (PD-1 or PD-L1) antibodies versus docetaxel alone in advanced non small cell lung cancer (NSCLC). In addition, the prognostic significance of PD-L1 expression in advanced NSCLC was also investigated. 5 eligible studies including 3579 patients were identified through comprehensive search of multiple databases. The results showed that pooled hazard ratios (HR) for overall survival (OS) and progression free survival (PFS) were 0.69 (95% CI: 0.63-0.75; *p* < 0.001) and 0.87 (95% CI: 0.80-0.94; *p* < 0.001), between PD-1/PD-L1 antibodies and docetaxel treatment arms, respectively. The pooled relative risk (RR) value for objective response rate (ORR) was 1.53, (95% CI: 1.16-2.01, *p* = 0.003). Further, subgroup analysis based on PD-L1 expression indicated that pooled HR for OS was significant with 0.66(95% CI: 0.59-0.74, *p* < 0.001) for PD-L1≥1%. However, PD-L1 < 1% had HR value of 0.79 (95% CI: 0.67-0.93, *p* = 0.006). Our study concluded that advanced NSCLC patients benefited more with PD-1/PD-L1 antibodies than docetaxel in the second line treatment. PD-L1≥10% in tumor tissues is sufficient to show significant improvement in patient's outcome with PD-1/PD-L1 antibodies compared to docetaxel. Moreover, PD-1/PD-L1 antibodies treatment showed significant decrease in conventional chemotherapy adverse events, but increased immune-associated adverse effects.

## INTRODUCTION

Lung cancer ranks first in term of causing cancer associated deaths worldwide, with an estimation of 221,200 new cases and 158,040 deaths alone in the year 2015 [1]. In United States, it accounts for 27% of all cancer related deaths in males, and 26% in females during the year 2016 [2], while in China, this is not only the most commonly diagnosed cancer and but also is the leading cause of cancer related deaths especially in men aged 75 years or older, and in women 60 years or older [3]. Lung cancer is usually classified into small cell lung cancer (15%) and non-small cell lung cancer (NSCLC) (85%). The NSCLC type has been further classified into non-squamous (NSQ) or squamous (SQ), and approximately 70% of the patients harbor a non-squamous histology. The NSQ subtype has further been categorized into adenocarcinoma (ADC), large cell carcinoma and adeno squamous carcinoma subgroups [4, 5]. Effective treatment options for NSCLC are still required due to very little progress in this direction since the approval of docetaxel as a second-line treatment in 1999 [6]. At present, targeted therapies such as tyrosine kinase inhibitors (TKI), antibodies against epidermal growth factor receptor (EGFR), inhibitors for anaplastic lymphoma kinase (ALK), and chemotherapy with platinum-based doublets have been the choice for first line treatment of advanced NSCLC. In addition, the docetaxel, a specific inhibitor of metaphase step in the cell cycle, has been a classic second-line therapeutic option [6–8]. However, in recent years, the development of immune checkpoint inhibitors have changed the treatment paradigm of advanced NSCLC. Recent studies have clearly revealed the mechanistic insight about the role of these checkpoint inhibitors as tumor suppressive targets. In peripheral tissues, the adaptive immune responses against tumor cells is negatively regulated, in part by binding of activated T cells expressing PD-1 with the PD-L1 and/or PD-L2 on tumor cells [8]. This, upregulated expression of PD-L1 on tumor cells helps to evade immune response [9], by inhibiting the T-cell responses and leads to immune resistance [10]. Thus, PD-1 and PD-L1 inhibitors/antibodies have been able to restore the T cells function in peripheral tissues by blocking the direct binding of T cell expressing PD-1 with PD-L1 or PD-L2 on tumor cells [10, 11]. In 2015, two PD-1 inhibitors/antibodies (nivolumab and pembrolizumab) have received US Food and Drug Administration (FDA) approval for the use in advanced NSCLC after platinum-based chemotherapy. Nivolumab has been recommended for patients with SQ-NSCLC and NSQ-NSCLC, while pembrolizumab for NSCLC tumors expressing PD-L1 [11–13].

Apparently the cost of immunotherapy for advanced NSCLC has been enormous [14] and is difficult for many patients in different countries to bear this cost. Therefore, an effective and less expensive treatment strategy is required to guide the application of immune checkpoint inhibitors/antibodies. There have been some studies indicating that PD-L1 expression level can serve as a biomarker for PD-1/PD-L1 antibodies based immunotherapy in NSCLC [15–20]. But there is an ambiguity in terms of PD-L1 expression being a suitable marker for PD-1/PD-L1 antibodies treatment for advanced NSCLC compared with docetaxel in the second-line treatment. To address this issue, we conducted a meta-analysis of randomized clinical trials (RCTs) to determine the efficacy and safety of PD-1 or PD-L1 antibodies compared with standard second-line therapy docetaxel alone and to assess the possible association between the level of PD-L1 and the prognosis of PD-1/PD-L1 antibodies in patients of advanced NSCLC.

## RESULTS

### Selection of studies and their characteristics

**Figure 1:**
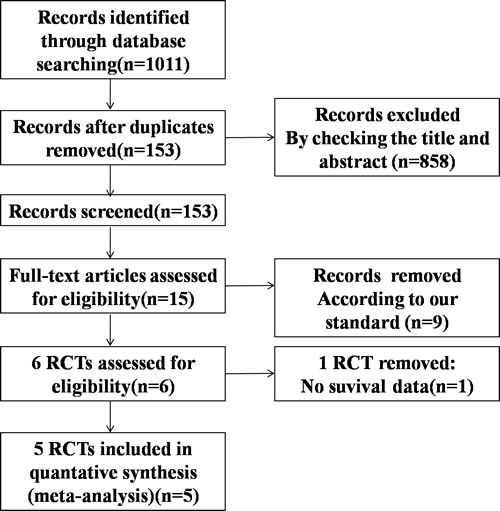
Flowchart depicting the RCTs selection process

**Figure 2:**
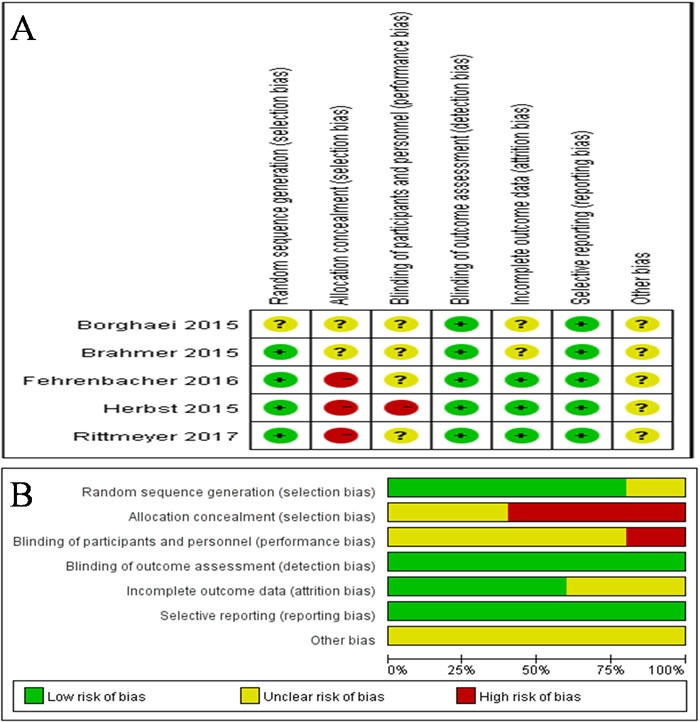
Risk of bias summary **A.** Risk of bias for each included RCT, representing low risk of bias (+), high risk of bias (-), and unclear risk of bias (?). **B.** Bar chart comparing percentage risk of bias for each included RCT. Low risk of bias (Green), high risk of bias (Red), and unclear risk of bias (Yellow).

Based on the search criteria, we initially identified 1011 studies from our database search. Among these, 858 studies were excluded because they did not fit our selection criteria. Of the remaining 153 studies, only 5 randomized controlled trials (RCTs) passed the inclusion criteria, and others were excluded because of repetitive or insufficient information (Figure 1). All the 5 included RCTs evaluated and compared the effectiveness of PD1/PD-L1 antibody therapies in advanced NSCLC over docetaxel, representing data from total of 3579 patients (Table 1 and 2). Out of this, 1851 patients were administered PD1/PD-L1 antibodies, while 1728 patients were given docetaxel. In addition, among the five studies, one had data from SQ-NSCLC patients [15], while another one had data from NSQ-NSCLC patients [16], and the remaining three studies [17, 18, 19] had data from both SQ and NSQ NSCLC patients. The study by Herbst *et al*. had analyzed two dosage (2 and 10 mg/kg) of pembrolizumab. Furthermore, the Cochrane risk of bias tool was used to measure the quality of the included studies, and the results are shown in Figure 2. Most of the included studies describe the detail of random sequence generation, Blinding of outcome assessment, incomplete outcome data and selective reporting. In some RCTs allocation was unmasked. Some studies did not mention allocation concealment, blinding of participants and personnel or random sequence generation. The other indexes of bias usually lacked specific description in the included clinical studies.

**Table 1:** Characteristics of the eligible RCTs included in the meta-analysis

study[year]	Study type	histology	endpiont	Treatment arms	Patients	CR+PR(%)	OS(m)	PFS(m)
Borghaei et al. [2015]	RCT III	NSQ	OS	nivolumab 3mg/kg q2w	292	56(19%)	12.2	2.3
				DOX 75mg/m2 q3w	290	36(12%)	9.4	4.2
Brahmer et al. [2015]	RCT III	SQ	OS	nivolumab 3mg/kg q2w	135	27(20%)	9.2	3.5
				DOX 75mg/m2 q3w	137	12(9%)	6.0	2.8
Fehrenbacher[2016]	RCT II	SQ and NSQ	OS	atezolizumab 1200mg q3w	144	21(14.6%)	12.6	2.7
				DOX 75mg/m2 q3w	143	21(14.7%)	9.7	3.0
Herbst et al. [2015]1	RCT III	SQ and NSQ	OS	pembrolizumab 2mg/kg q2w	344	62(18.0%)	10.4	3.9
				DOX 75mg/m2 q3w	343	32(9.3%)	8.5	4.0
Herbst et al. [2015]2	RCT III	SQ and NSQ	OS	pembrolizumab 10mg/kg q2w	346	64(18.5%)	12.7	4.0
				DOX 75mg/m2 q3w	343	32(9.3%)	8.5	4.0
Rittmeyer et al.[2017]	RCT II	SQ and NSQ	OS	atezolizumab 1200mg q3w	425	58(13.6%)	13.8	2.8
				DOX 75mg/m2 q3w	425	57(13.4%)	9.6	4.0

RCT: randomized controlled trials; SQ: Squamous non small cell lung cancer; NSQ: Non-squamous non small cell lung cancer; DOX: docetaxel

**Table 2:** Treatment-Related AEs (Grade 1-4/3-4) for PD-1/PD-L1 antibodies vs. docetaxel

AEs (Grade 1–4/3-4)	PD1/PD-L1antibody	DOX	Heterogeneity P and I^2^		RR (95%CI)	Z value	*P* value
any events(G1-4)	1201/1851	1464/1728	0.421	0.0%	0.77(0.74,0.79)	13.38	0.000
(G3-4)	284/1851	751/1728	0.000	91.0%	0.33(0.22,0.51)	5.03	0.000
Nausea(G1-4)	239/1851	358/1728	0.047	55.0%	0.58(0.46,0.75)	4.28	0.000
(G3-4)	10/1851	8/1728	0.827	0.0%	0.15(0.48,2.77)	0.31	0.756
Febrile neutropenia(G1-4)	1/1851	146/1728	0.994	0.0%	0.02(0.01,0.06)	7.06	0.000
(G3-4)	1/1851	144/1728	0.994	0.0%	0.02(0.01,0.07)	7.03	0.000
Diarrhea(G1-4)	182/1851	371/1728	0.032	59.0%	0.41(0.31,0.55)	5.98	0.000
(G3-4)	9/1851	35/1728	0.800	0.0%	0.26(0.13,0.52)	3.79	0.000
Neutropenia(G1-4)	16/1851	322/1728	0.051	55.0%	0.04(0.02,0.10)	6.74	0.000
(G3-4)	3/1851	246/1728	0.684	0.0%	0.02(0.01,0.05)	9.04	0.000
Anemia(G1-4)	110/1851	319/1728	0.001	77.0%	0.25(0.14,0.42)	5.01	0.000
(G3-4)	19/1709	54/1593	0.658	0.0%	0.34(0.20,0.56)	4.17	0.000
Fatigue(G1-4)	354/1851	524/1728	0.225	28.0%	0.63(0.56,0.71)	7.65	0.000
(G3-4)	32/1851	72/1728	0.281	20.0%	0.42(0.28,0.63)	4.17	0.000
Rash(G1-4)	105/1100	44/1015	0.070	57.0%	2.01(1.14,3.51)	2.43	0.020
(G3-4)	3/1100	2/1015	0.540	0.0%	1.17(0.31,4.42)	0.24	0.810
Alopecia(G1-4)	11/1851	551/1728	0.900	0.0%	0.02(0.01,0.04)	13.31	0.000-
(G3-4)	0/1851	7/1728	0.997	0.0%	0.25 (0.06,0.99)	1.98	0.048
Colitis(G1-4)	11/1242	0/1150	0.999	0.0%	4.99 (1.45,17.11)	2.55	0.011
(G3-4)	7/1242	0/1150	0.994	0.0%	3.55 (0.88,14.28)	1.78	0.075
Hypothyroidism(G1-4)	87/1242	2/1150	0.974	0.0%	23.36(8.04-67.90)	5.79	0.000
Hyperthyroidism(G1-4)	36/969	6/886	0.765	0.0%	5.10(2.23-11.68)	3.85	0.000
Pneumonitis(G1-4)	62/1242	18/1150	0.653	0.0%	3.19(1.90-5.34)	4.40	0.000
interstitial lung disease(G1-4)	5/1100	5/1015	0.607	0.0%	0.93(0.29-2.87)	0.13	0.893

### Overall survival analysis

**Figure 3:**
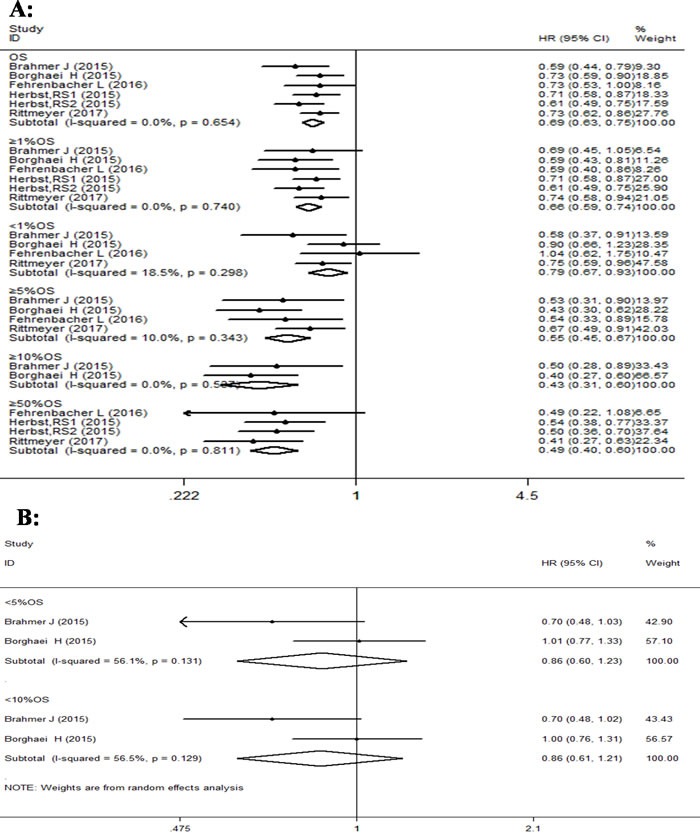
Forest plot analysis for OS between patients treated with PD-1/PD-L1 antibodies and docetaxel monotherapy along with different levels of PD-L1 expression **A**. (I-squared < 50%, FEM): All patients, PD-L1≥1%, PD-L1 < 1%, PD-L1≥5%, PD-L1≥10%, PD-L1≥50%; **B**. ( I-squared ≥50%, RAM): PD-L1 < 5%, PD-L1 < 10%.

The forest plot analysis of overall survival with PD-1/PD-L1 antibodies indicated better prognosis than docetaxel, in advanced NSCLC patients, as shown in Figure 3. Compared with docetaxel, we observed a significant decrease (31%) in the risk of death in PD-1/PD-L1 antibody group (HR 0.69, 95% CI: 0.63-0.75, p < 0.001; I^2^ = 0%). Further subgroup analysis of OS based on PD-L1 expression again revealed statistically significant advantage for PD-1/PD-L1 therapy as compared to docetaxel, with pooled HR values of 0.79 (95% CI: 0.67-0.93, p = 0.006) in subgroups with PD-L1 expression of < 1%,0.66 (95% CI: 0.59-0.74, p < 0.001) with PD-L1 expression of ≥1%, 0.55 (95% CI: 0.45-0.67, p < 0.001) with PD-L1 expression of ≥5%, 0.41 (95% CI: 0.27-0.63, p < 0.001) with PD-L1 expression of ≥10%, and 0.49 (95% CI: 0.40-0.60, p < 0.001) with PD-L1 expression of ≥50%. However, the pooled HR values were not statistically significant in subgroups with PD-L1 expression of < 5% [0.86(95% CI: 0.61-1.23, p = 0.417)], and < 10% [0.86(95% CI: 0.61-1.21, p = 0.381)]. In addition, we found very little overall heterogeneity for OS in all studies (I^2^ = 0%, p = 0.654), but the heterogeneity at the PD-L1 expression subgroup levels was different. For instance, PD-L1 expression of ≥1%, 5%, 10%, 50% and < 1 %, displayed I^2^ values of 0% (p = 0.740); 10.0% (p = 0.343); 0% (p = 0.537); 0% (p = 0.811);18.5% (p = 0.298). respectively, and represented less heterogeneity. However other subgroups based on PD-L1 expression like, < 5% and < 10% showed I^2^ values of 56.1% (p = 0.131) and 56.5% (p = 0.129), respectively, and suggested high heterogeneity (Figure 3A & 3B).

### Progression free survival analysis

**Figure 4:**
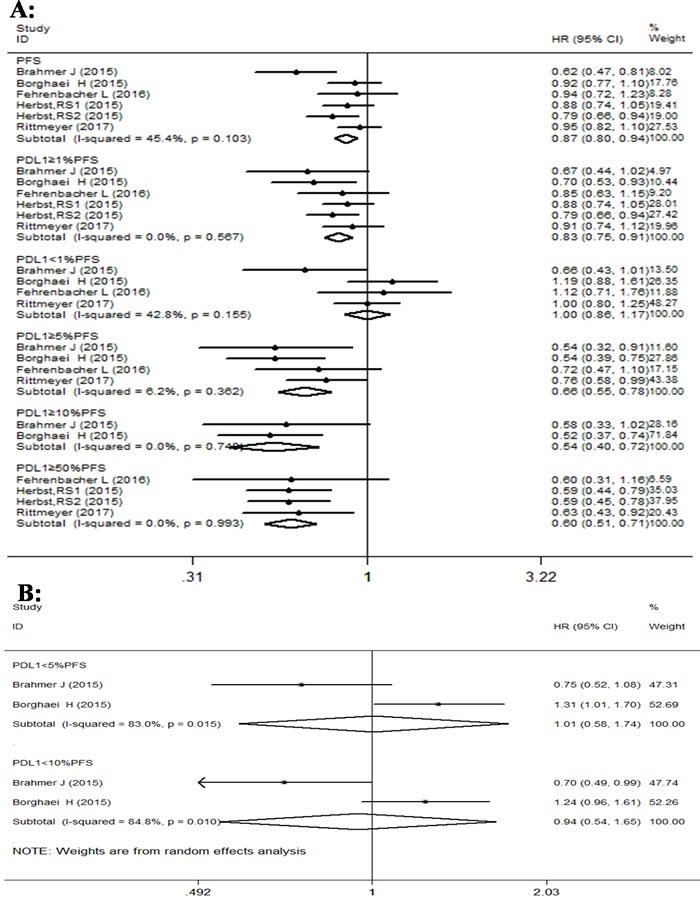
Forest plot analysis for PFS between patients treated with PD-1/PD-L1 antibodies and docetaxel monotherapy along with different levels of PD-L1 expression **A**. (I-squared < 50%, FEM): All patients, PD-L1≥1%, PD-L1≥5%, PD-L1≥10%, PD-L1≥50%. **B**. ( I-squared ≥50%, REM): PD-L1 < 1%, PD-L1 < 5%, PD-L1 < 10%.

Similarly, forest plot analysis of PFS indicated better results with PD-1/PD-L1 antibodies than docetaxel in advanced NSCLC patients (Figure 4). The PD-1/PD-L1 antibodies displayed significant improvement in PFS of advanced NSCLC patients, with HR value of 0.87 (95% CI: 0.80-0.94; p < 0.001). The subgroup analysis for PFS based on PD-L1 expression also showed statistically significant improvement in some subgroups with PD-1 antibody treatment over docetaxel. The pooled HR values of subgroups with PD-L1 expression of ≥1%, 5%, 10% and 50% were 0.83 (95% CI: 0.75-0.91, p = 0.000); 0.65 (95% CI: 0.55-0.79, p < 0.001); 0.54 (95% CI: 0.40-0.72, p < 0.001); and 0.59 (95% CI: 0.51-0.71, p < 0.001), respectively. However, the pooled HR values of subgroups with PD-L1 expression of < 1%, < 5% and < 10% were 1.00 (95% CI: 0.86-1.17, p = 0.968); 1.01 (95% CI: 0.58-1.74, p = 0.982); and 0.94 (95% CI: 0.54-1.65, p = 0.839), respectively, and did not show statistically significant improvement. Overall, there was some heterogeneity for PFS in all studies (I^2^ = 45.4%, p = 0.103). The subgroup analysis for PFS based on PD-L1 expression showed different levels of heterogeneity, ranging from no to significant levels. For instance, PD-L1 expression with ≥1%, 5% ,10%, 50% and < 1% showed I^2^ values of 0% (p = 0.567); 6.2% (p = 0.362); 0% (p = 0.748); 0% (p = 0.993) and 42.8% (p = 0.155), respectively. But PFS analysis of PD-L1 subgroups with expression < 5% and < 10% showed I^2^ values of 83% (p = 0.015) and 84.8% (p = 0.010), respectively, and represented high heterogeneity.

### ORR analysis

**Figure 5:**
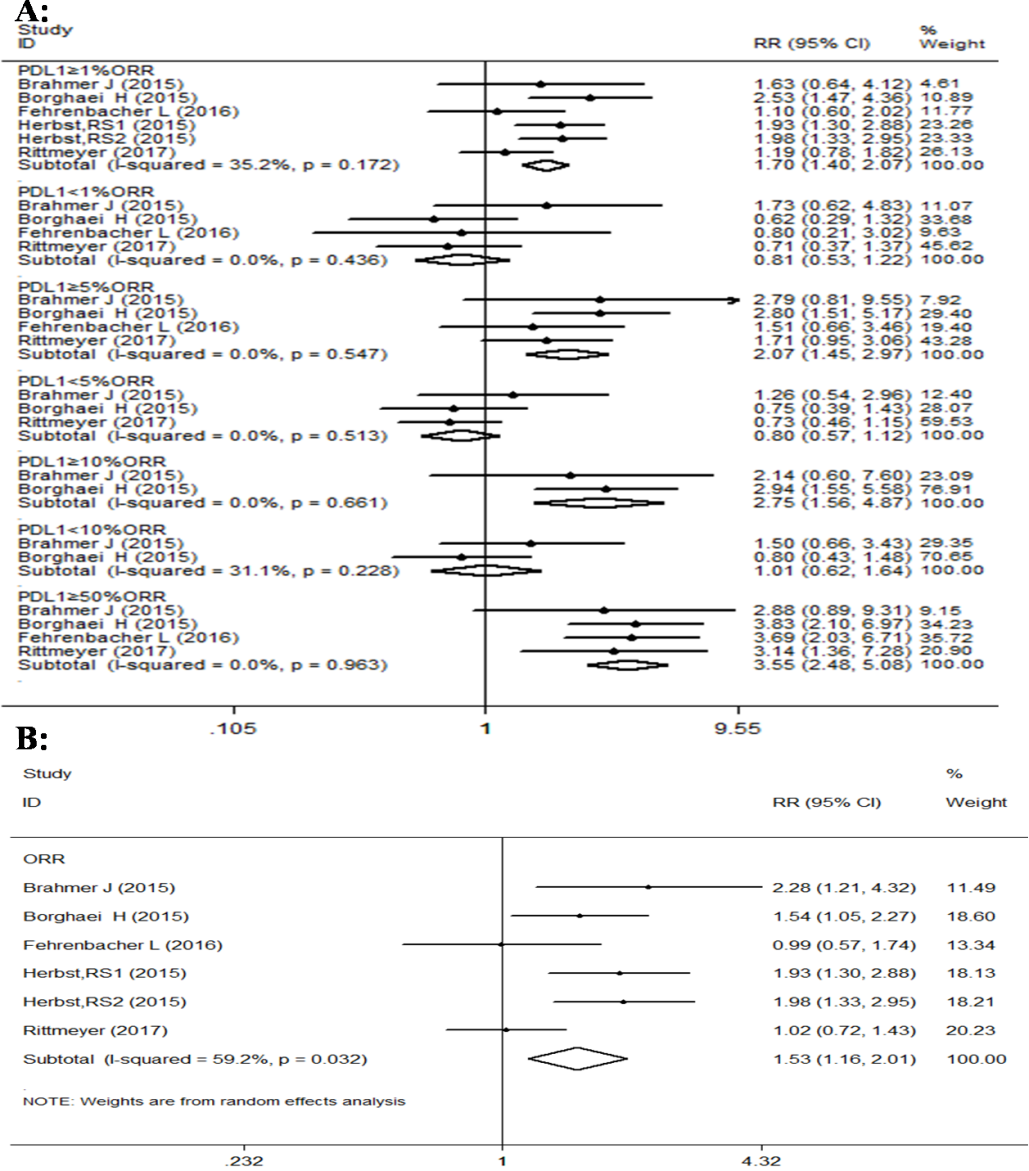
Forest plot analysis for ORR between patients treated with PD-1/PD-L1 antibodies and docetaxel monotherapy along with different levels of PD-L1 expression **A**. (I-squared < 50%,FEM):PD-L1≥1%,PD-L1≥5%,PD-L1≥10%,PD-L1≥50%,PD-L1 < 1%,PD-L1 < 5%,PD-L1 < 10%; **B**. ( I-squared ≥50%, RAM): All patients.

The comparison of overall response rate in all four RCTs also demonstrated that PD-1/PD-L1 antibodies have significantly better effect than docetaxel (Figure 5). The ORR analysis which was done using fixed effects model due to very little heterogeneity between the different trials, except overall RR value of 1.53, (95% CI: 1.16-2.01, P = 0.003; I^2^ = 59.2%) in favor of PD-1/PD-L1 antibodies in NSCLC patients. Further subgroup analysis of ORR according to PD-L1 expression also indicated that the pooled RRs in subgroups were in favor of PD-1 antibodies treatment. For example subgroups displaying PD-L1 expression of ≥1%, 5%,10% and 50% had RR values of 1.70 (95% CI: 1.40-2.07, p < 0.001); 2.08 (95% CI: 1.45-2.97, p < 0.001); 2.75 (95% CI: 1.56-4.87, p = 0.001); and 3.55 (95% CI: 2.48-5.08, p < 0.001), respectively. However, the pooled RRs of subgroups with PD-L1 expression of < 1%, < 5% and < 10% were 0.81 (95% CI: 0.53-1.22, p = 0.305); 0.79(95% CI: 0.57-1.12, p = 0.196); and 1.01 (95% CI: 0.62-1.64, p = 0.978), respectively, and were not statistically significant. The heterogeneity among other subgroups except overall RR was between 0 to 50% and was thus not a major factor effecting RR values.

### Adverse events analysis

As compared with standard second line docetaxel chemotherapy, the advanced stage NSCLC patients receiving PD-1/PD-L1 antibodies showed significant increase in the incidence rate of grade 1-4 adverse events (AEs). The overall RR value for AE was 0.77 (95% CI: 0.74-0.79; P = 0.000), and specific RRs based on each event also showed significant effect, like for rashes, the RR value was 2.01, 95% CI: 1.14-3.51, P = 0.02; for hypothyroidism, the RR value was 23.36, 95% CI: 8.04-67.90; P < 0.001; for hyperthyroidism, the RR value was 5.1, 95% CI: 2.23-11.68, P < 0.001; for pneumonitis, the RR value was 3.19, 95% CI: 1.9-5.34, P < 0.001; and for colitis, the RR value was 4.99, 95% CI: 1.45-17.11, P = 0.011. But, we did not observe any significant difference for the incidence rate of interstitial lung disease between both groups (Table 2). However, compared with docetaxel, the patients receiving PD-1/PD-L1 antibodies showed significant decrease in the incidence rate of grade 1-4 AEs such as nausea, febrile neutropenia, diarrhea, anemia, neutropenia, fatigue and alopecia (nausea: RR 0.58, 95% CI: 0.46-0.75, P < 0.001; febrile neutropenia: RR 0.02, 95% CI: 0.01-0.06, P < 0.001; diarrhea: RR 0.41, 95% CI: 0.31-0.55; neutropenia: RR 0.04, 95% CI: 0.02-0.10, P < 0.001; anemia: RR 0.25, 95% CI: 0.14-0.42, P < 0.001; fatigue: RR 0.63, 95% CI: 0.56-0.71, P < 0.001; alopecia: RR 0.02, 95% CI: 0.01-0.04, P < 0.001).

In addition we also compared the grade 3-4 adverse events between PD-1/PD-L1 antibody and docetaxel alone treatment arms. The patients receiving PD-1/PD-L1 antibodies showed significant decrease in grade 3-4 AEs with overall RR value of 0.33; 95% CI: 0.22-0.51, P < 0.001. Specifically febrile neutropenia, diarrhea, neutropenia, anemia and fatigue displayed following RR values(febrile neutropenia: RR 0.02, 95% CI: 0.01-0.07, P < 0.001; diarrhea: RR 0.26, 95% CI: 0.13-0.52; neutropenia: RR 0.02, 95% CI: 0.01-0.05, P < 0.001; anemia: RR 0.34, 95% CI: 0.20-0.56, P < 0.001; fatigue: RR 0.42, 95% CI: 0.28-0.63, P < 0.001; alopecia: RR 0.25, 95% CI: 0.06-0.99, P = 0.048). However the incidence rates of other grade 3-4 AEs including: nausea, rash and colitis, did not show any significant difference between PD-1 antibodies and docetaxel therapy (Table 2).

### Analysis of publication bias

To evaluate the publication bias between different studies, STATA 12.0 software based Egger's test and Begg's test were utilized. The results have been presented in Table 3, and all P values were more than 0.05 after both tests, thereby, suggesting no significant publication bias in our meta-analysis.

**Table 3:** Evaluation of publication bias with Begg's test and Egger's test

	trails	Heterogeneity		HR/RR (95%CI)	Begg's test		Egger's test	
		*P*	*I*^2^		Z	*P*	T	*P*
**OS**	5	0.654	0.0%	0.69(0.63,0.75)	1.13	0.26	-0.89	0.422
**PFS**	5	0.103	45.0%	0.87(0.80,0.94)	1.13	0.26	-1.34	0.252
**ORR**	5	0.032	59.0%	1.53(1.16,2.01)	1.32	0.19	0.66	0.546
**AEs(G1-4)**	5	0.421	0.4%	0.77(0.74,0.80)	0.75	0.45	-0.60	0.582
**AEs(G3-4)**	5	0.000	91.0%	0.33(0.22,0.51)	0.75	0.45	-0.82	0.458

## DISCUSSION

PD-1 and PD-L1 molecules play an important role in limiting the tumor suppressive function of T cells. Upregulated PD-L1 on tumor cells binds to PD-1 on T cells, and turn off the immune surveillance. Consequently, tumor cells escape the body immune response against them and have unlimited growth [21]. Recently, the checkpoint inhibitors/antibodies, such as PD-1/PD-L1 antibodies have been observed to enhance the tumor suppressor activity of T immune cells in solid tumors including advanced NSCLC, and have shown very promising results in multiple cancers in many clinical trials. Herein, we have selected 5 RCTs including NSCLC patients to perform the meta-analysis to compare the efficacy of PD1/PD-L1 antibodies with docetaxel. These RCTs have alternatively been referred as CheckMate017, CheckMate057, KEYNOTE010, POPLAR and OAK [15–19]. Many additional clinical trials of PD-1/PD-L1 antibodies are also ongoing such as KEYNOTE-042 which intend to compare pembrolizumab as first-line therapy against platinum doublet chemotherapy for advanced NSCLC [22–24].

Our meta analysis showed that PD-1/PD-L1 antibodies treatment significantly improved the OS, PFS and ORR of advanced SQ- and NSQ-NSCLC patients as compared to docetaxel as second-line therapy. Moreover, the OS, PFS and ORR were significantly elevated with PD-1 or PD-L1 antibodies than docetaxel in subgroups with PD-L1 expression of ≥1%, 5%, 10% and 50%. because subgroups with PD-L1 expression less than 5%, 10% did not show significant differences. Thus, According to our meta analysis, we concluded that PD-L1 expression of ≥10% on tumor cells, can be a cutoff value for guiding the application of PD1/PDL1 antibodies therapy in NSCLC patients. As can be seen, PD-L1 expression of ≥50% on tumor cells has supported the application of PD1/PDL1 antibodies therapy in NSCLC patients. Latest NCCN 2017 of lung cancer also bring forword PD-L1 expression of ≥50% is a cutoff value for guiding the application of PD1/PDL1 antibodies therapy in NSCLC patients in 1^st^ line. However, different studies have reported different PD-L1 cutoff values to be considered as PD-L1 positive [25, 26]. Notably, membranous and/or cytoplasmic PD-L1 expression in lung cancer cells was assessed by immunohistochemistry (IHC) in CheckMate017, CheckMate057 and KEYNOTE010 RCTs [15, 16, 18], but now some studies in various solid cancers have indicated that combined PD-L1 expression on tumor infiltrating lymphocytes and dendritic cells (TILs and TIDCs) in the cancer tumor microenvironment could be important [27–29]. In the RCTs analyzed in our study, the evaluation of PD-L1 expression appears more complex in two RCTs (POPLAR and OAK) than other 3 RCTs, because it considered PD-L1 expression from both tumor cells and infiltrating immune cells [17, 19].

Although PD-1/PD-L1 inhibitors/antibodies are a better treatment option, but due to their higher cost, they are less accessible to many patients. Thus identification of a markers which can enable the patients to figure out the possibility of effective treatment with these checkpoint inhibitors, would go a long way in helping them to invest their resources wisely. Regarding the cost-effectiveness, it has been suggested that PD-L1 expression of ≥1% improved incremental quality-adjusted life-years (QALY) with nivolumab, for patients with NSQ tumors by 67%, and led to 40% reduction in the incremental costeffectiveness ratios (ICER) (from US$ 176K to 105K). In case of pembrolizumab, the use of 50% instead of 1% cutoff, helped to increase the incremental QALY by 18% and reduced ICER by 15% (from US Dollar163K to 138K) [14]. Consistent with these suggestions, our analysis, showed that PD-L1expression cutoff of 10% was sufficient to display the advantage of choosing PD-1/PD-L1 antibody therapy in NSCLC over standard second line chemotherapy, docetaxel.

However, in our meta, the OS of the patients was significantly elevated with PD-1/PD-L1 antibodies than docetaxel in subgroups with PD-L1 expression of < 1%, which is consistant with the rusults of several some studies [30–32]. Brahmer [15] did not suggest PD-L1 expression as an effective biomarker of selecting patients for PD-1/PD-L antibody therapy, especially when those with tumors lacking PD-L1 expression (PD-L1 < 1%) has also been observed to derive benefit from PD-1/PD-L1 antibodies in advanced NSCLC. In fact, there exist several challenges in using PD-L1 expression as a powerful indicator for choosing the PD/PDL therapy. Firstly, the expression of PD-L1 shows much heterogeneity of an individual, which might reflects the heterogenous expression of PD-L1 among cancerous cells in the primary tumor, the differenc of PD-L1 expression between the primary lesion and its metastases, and finally, the difference of PD-L1 expression of the tumor and its surrounding stoma cells, especially inflammatory cells such as lymphocytes and macrophages [33–36]. Secondly, PD-L1 also manifests dynamic, but not static expression manner in the patients, which, for example, can be induced by activated tumor-specific T cells [34, 37]. Thirdly, immunohistochemistry is the mostly employed to determine the positivity of the expression of PD-L1, which could be confined by the availablity and efficency of its current commercial anibodies. At least, 5 antibodies, Dako 28-8, Dako 22C3, Roche ventana, SP142 and SP263 are used in the clinical trials for PD-1/PD-L1therapy [38]. How to understand and bridge the divergence of the positive rate determined by different antibodies constitutes a technical chanllenge for pathologists. Now, A more accurate, quantificable and objective method for determining the PD-L1 positive rate is in urgent need in clinic, where those such as examination of PD-L1 positive circulating tumor cells with flow cytometry and mismatch-repair deficiency(MMR) hold promise [39, 40].

The adverse events due to immunotherapy have been because of disruption in immune tolerance. The immune-related adverse events (irAEs) are usually defined as any AE associated with drug exposure and consistent with an immune-mediated mechanism of action [41, 42]. Our analysis indicated that anti PD-1 therapy is associated with fewer adverse events than docetaxel treatment, which is in accordance with previous reports about grade 1-4 nausea, febrile neutropenia, diarrhea, anemia, neutropenia, fatigue and alopecia. Endocrinopathies are perhaps the most elusive irAEs, due to their nonspecific presentations. Among these, thyroid changes (mostly hypothyroidism) are the most common endocrine events, reported in about 3%-6% of NSCLC patients treated with PD-1 checkpoint inhibitors. Hypothyroidism can still be found in asymptomatic patient, and this could involve elevated levels of thyroid-stimulating hormone (TSH) or even without it. In addition, presentations of hypothyroidism, hyperthyroidism and pneumonitis due to PD-1/PD-L1 antibodies are 23.36, 5.10 and 3.19 folds higher than docetaxel group (P < 0.001). The off-target effects against the normal lung parenchyma could be a result of increased immune response against the tumor by PD-1inhibition. Although, anti-PD-1 drugs rarely caused pneumonitis (any grade 4-6%, grade 3-4: 0-2%), but pneumonitis has significant potential for morbidity and mortality, and thus, occurence of it should be approached with caution. Similar to fatal pneumonitis, interstitial lung disease (ILD) have also been represented in advanced NSCLC patients treated with EGFR-TKIs (gefıtinib: 3.5%; erlotinib: about 1.6%-4.5%) [43, 44] and with chemotherapy (docetaxel: 4.6%; gemcitabine: < 1%) [45–47]. In recent years, there has been reports about docetaxel-related interstitial lung disease (ILD), where ILD onset occurred 10-20 days (median time: 18 days) after docetaxel administration [48, 49]. However, in our meta-analysis, we did not find any difference in ILD between patients treated with PD-1/PD-L1 antibodies or docetaxel.

Thus, in conclusion, our meta-analysis study indicated that PD-1/PD-L1 antibodies treatment indeed has beneficial effects on advanced NSCLC patients in comparison to docetaxel monotherapy, along with displaying few adverse events. In addition, the PD-L1 expression of more than 10% on tumor tissues can serve as a biomarker to identify the NSCLC patient populations that might respond positively to PD-1/ PD-L1 antibody therapy, and thereby helping many patients to make a informed decision about this high cost immunotherapy.

## MATERIALS AND METHODS

### Search strategy

All the random controlled trials with information about NSCLC, docetaxel, PD1 and PD-L1 antibodies, from January 1990 to January 2017, were searched using the following databases, Cochrane library, Embase, PubMed, China hospital knowledge database, China National Knowledge Infrastructure, Wangfang Data and Weipu Data. The medical subject heading (MeSH) terms included for searching the relevant studies were: lung neoplasm, pulmonary neoplasm, lung carcinoma, pulmonary carcinoma, lung cancer, pulmonary cancer and PD-1 or PD-L1, PD-1 or PD-L1 inhibitors and chemotherapy or docetaxel.

### Inclusion critera

The studies with the following information were selected for inclusion in our meta-analysis; (1) Phase II/III (randomized controlled trails) RCTs with primary endpoints as OS or PFS; (2) histological confirmed SQ and/or NSQ non small cell lung cancer; (3) having the information about OS, PFS and ORR, AEs, and PD-L1 expression; (4) published in English language; and (5) having some similarity between experimental design and methods across different studies. However, the studies were excluded if they were: (1) reviews, duplicate reports, letters, unfinished studies, or conference reports; (2) studies conducted with cell lines, animal models or other types of non-lung cancers; (3) studies where HR and 95% CI could not be determined due to insufficient survival data; (4) papers in other laungages except English; (5) methods or experimental design were substantially different from other selected RCTs; and (6) sample size was smaller than 100.

### Data extraction

Two reviewers (Qiang Su and Yanli Hou) independently searched all the relevant studies and read the titles, abstracts and full texts of the identified studies. Cases of disagreement were resolved through discussion with the third reviewer (Hongchao Zhen). The following information was extracted from the selected studies; year of publication, name of the journal, author's name, methods of randomization, OS, PFS, ORR, PD-L1 expression rate and adverse events (AEs) with grades 1-4 and 3/4.

### Data analysis

In our meta-analysis, Risk of bias analysis was prepared using Review Manager 5.3 software (Cochrane Collaboration 2014,Nordic Cochrane Center, Copenhagen, Denmark). Two reviewers (Q.S. and C.G.Z.) independently assessed the quality of the included studies according to the Cochrane risk of bias tool, which assesses the following six domains: selection bias (including both “Random sequence generation” and “allocation concealment”), performance bias, detection bias, attrition bias, reporting bias and other bias. The Stata version 12.0 statistical software (Stata Corporation, College Station, Texas, USA) was used to conduct the meta-analysis. The HR and 95% CI values were collected and merged to estimate overall OS and PFS. The HR value of < 1.0 indicated reduced progression or death in the PD-1/PD-L1 antibody group. The RR value was used to estimate ORR and AEs for grade 1-4 /3-4, and RR value of >1.0 represented higher ORR or the incidence of grade 1-4 and 3/4 AEs in the PD-1/PD-L1 antibodies group. In addition, the Cochran's χ^2^ test was used to assess the heterogeneity among the RCTs. When I^2^ value was < 50%, the fixed-effects model (FEM) was employed for analysis, and if I^2^ value was ≥50%, random-effects model (REM) was used. The Begg's and Egger's tests were used to analyze the publication bias between different RCTs.
